# Three‐dimensional modelling identifies novel genetic dependencies associated with breast cancer progression in the isogenic MCF10 model

**DOI:** 10.1002/path.4778

**Published:** 2016-10-19

**Authors:** Sarah L Maguire, Barrie Peck, Patty T Wai, James Campbell, Holly Barker, Aditi Gulati, Frances Daley, Simon Vyse, Paul Huang, Christopher J Lord, Gillian Farnie, Keith Brennan, Rachael Natrajan

**Affiliations:** ^1^ The Breast Cancer Now Toby Robins Research Centre, Division of Breast CancerThe Institute of Cancer ResearchLondonUK; ^2^ Division of Molecular PathologyThe Institute of Cancer ResearchLondonUK; ^3^ Division of Cancer BiologyThe Institute of Cancer ResearchLondonUK; ^4^ Institute of Cancer SciencesUniversity of ManchesterManchesterUK; ^5^ Faculty of Life SciencesUniversity of ManchesterManchesterUK

**Keywords:** breast cancer progression, 3D spheroid assays, next‐generation sequencing

## Abstract

The initiation and progression of breast cancer from the transformation of the normal epithelium to ductal carcinoma in situ (DCIS) and invasive disease is a complex process involving the acquisition of genetic alterations and changes in gene expression, alongside microenvironmental and recognized histological alterations. Here, we sought to comprehensively characterise the genomic and transcriptomic features of the MCF10 isogenic model of breast cancer progression, and to functionally validate potential driver alterations in three‐dimensional (3D) spheroids that may provide insights into breast cancer progression, and identify targetable alterations in conditions more similar to those encountered in vivo. We performed whole genome, exome and RNA sequencing of the MCF10 progression series to catalogue the copy number and mutational and transcriptomic landscapes associated with progression. We identified a number of predicted driver mutations (including PIK3CA and TP53) that were acquired during transformation of non‐malignant MCF10A cells to their malignant counterparts that are also present in analysed primary breast cancers from The Cancer Genome Atlas (TCGA). Acquisition of genomic alterations identified MYC amplification and previously undescribed RAB3GAP1–HRAS and UBA2–PDCD2L expressed in‐frame fusion genes in malignant cells. Comparison of pathway aberrations associated with progression showed that, when cells are grown as 3D spheroids, they show perturbations of cancer‐relevant pathways. Functional interrogation of the dependency on predicted driver events identified alterations in HRAS, PIK3CA and TP53 that selectively decreased cell growth and were associated with progression from preinvasive to invasive disease only when cells were grown as spheroids. Our results have identified changes in the genomic repertoire in cell lines representative of the stages of breast cancer progression, and demonstrate that genetic dependencies can be uncovered when cells are grown in conditions more like those in vivo. The MCF10 progression series therefore represents a good model with which to dissect potential biomarkers and to evaluate therapeutic targets involved in the progression of breast cancer. © 2016 The Authors. The Journal of Pathology published by John Wiley & Sons Ltd on behalf of Pathological Society of Great Britain and Ireland.

## Introduction

The initiation and progression of breast cancer from the transformation of the normal epithelium to carcinoma *in situ* and invasive disease is a multifaceted process that results in the acquisition of multiple genomic alterations, including changes in genomic copy number, structural rearrangements, acquisition of mutations, altered gene expression, and pathway dysregulation 1, 2, 3, 4. The transition through these states, i.e. non‐invasive to invasive disease, is a well‐defined and staged process, through which breast cancers progress to acquire the capacity to grow, persist, and eventually spread to secondary sites.

High‐throughput molecular profiling of breast cancers and their precursor lesions has revealed that they have distinct genomic and transcriptomic alterations 3, 5, 6, 7, 8; however, matched preinvasive lesions and invasive counterparts from the same patient are remarkably similar 6, 7, 8, 9, 10, suggesting that the extent of genomic heterogeneity is determined early in breast cancer development. There is evidence suggesting that the progression from *in situ* to invasive disease is not exclusively driven by specific genomic aberrations in the preinvasive cells, but is a result of paracrine interactions of tumour cells with the surrounding stromal environment 3, 11, 12, 13.

**Figure 1:**
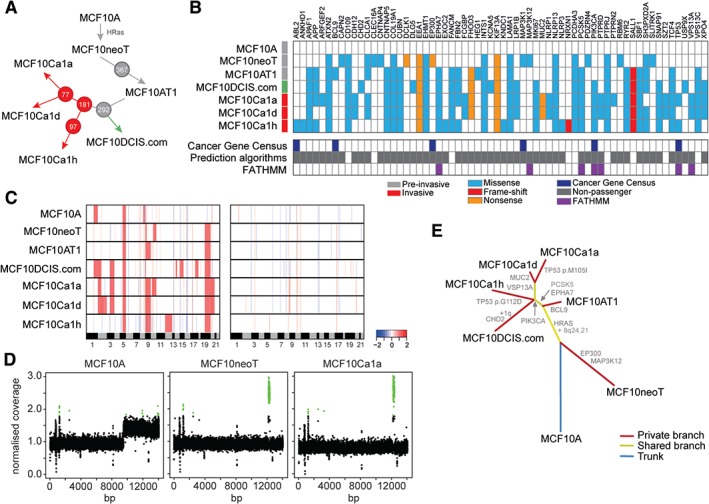
Spectrum of acquired alterations in the MCF10 progression series. (A) Diagrammatic representation of the generation of the MCF10 progression series. Non‐invasive cell lines are highlighted in grey, DCIS.com cell are highlighted in green, and invasive cell lines are highlighted in red. Circled numbers represent the number of days for which cell lines were grown in vivo before replantation. (B) Matrix of identified somatic mutations acquired from MCF10A cells that also occur in TCGA breast cancer data. Mutations were classified according to membership of Cancer Gene Census (navy blue) 79, the results of the gene driver prediction algorithm FATHMM (purple) 61 and other prediction algorithms (grey) are shown (see Materials and methods). (C) Heatmap of gains (red) and losses (blue) identified from GISTIC. The genomic position is plotted along the x‐axis, and samples are plotted on the y‐axis. A heatmap of focal (<10 Mb) amplifications and homozygous deletions identified from GISTIC is shown. The colour scale bar depicts homozygous deletions to amplifications (–2 to +2). Note the presence of focal amplification of MYC (8q24.21) acquired from MCF10neoT cells onwards. (D) Chromosome 8 plots of MCF10A, MCF10neoT and MCF10Ca1a cells of log_2_‐normalized sequencing reads (y‐axis) plotted against base pair position cross chromosome 8. Green represents an amplification log_2_ ratio of >1.8. (E) Unrooted phylogenetic tree generated by neighbour joining of the MCF10 progression series based on variant data and whole arm chromosomal changes acquired from MCF10A cells onwards. Driver mutations and chromosomal changes acquired are indicated in grey.

The MCF10 progression series is a product of the ‘normal’ mammary epithelial cell line MCF10A that is spontaneously immortalised from the MCF10 mortal cell line (MCF10M), which originated from benign fibrocystic disease 14. As MCF10A cells are non‐tumorigenic, cells were HRAS‐transformed to produce MCF10neoT and MCF10AT1 cells 15, 16 (Figure 1A). MCF10AT1 cells were subsequently serially passaged *in vivo* to produce carcinoma *in situ* MCF10DCIS.com 17 and the invasive carcinoma cells MCF10Ca1a, MCF10Ca1d and MCF10Ca1h 18, 19. MCF10Ca1a and MCF10Ca1d are *in vitro* clones derived from the same *in vivo* tumour, whereas MCF10Ca1h is derived from a separate tumour (Figure 1A). This series of cell lines therefore represents an isogenic model of disease progression, and provides a useful tool for the investigation of molecular changes during the progression of human breast neoplasia and the generation of tumour heterogeneity on a common genetic background 19.

Numerous studies have characterised different cell lines from the MCF10 progression series through the use of genomic, transcriptomic and proteomic profiling 20, 21, 22, 23, 24, 25, 26. These have shown that alterations that differ between the cell lines can identify drivers of different stages of breast cancer progression. Indeed, proteomic profiling has identified increased expression of AKT and STAT signalling in the invasive cell lines, events that are also known to occur in primary disease 26. Similar studies also identified secreted biomarkers that are known to be involved in metastasis of non‐invasive and invasive cells 27. The model has also proven useful in dissecting the role of poor prognostic biomarkers, such as BRMS1 and FSP1, *in vitro* 22, 28, and for the identification and functional assessment of novel biomarkers of progression from ductal carcinoma *in situ* (DCIS) to invasive disease with both three‐dimensional (3D) culture and *in vivo* models 21, 23, 29.

Here we sought to: (1) define the genomic characteristics, at base pair resolution, of the MCF10 breast cancer progression series of cell lines that are associated with different stages of progression; (2) determine the enrichment of pathway alterations in the progression from preinvasive to invasive disease; (3) establish an *in vitro* functional screening tool using cancer cell line spheroids, which more accurately recapitulate *in vivo* models; and (4) use this platform as biological proof of concept to identify potential driver genetic alterations of breast cancer progression.

## Materials and methods

### Cell lines

The isogenic MCF10 cell line series includes the initial untransformed normal cell line, MCF10A, the benign proliferation stages (MCF10AT1 and MCF10NeoT), the carcinoma *in situ* stage (MCF10DCIS.com), and the invasive carcinoma stages (MCF10Ca1a cl1, MCF10Ca1d cl1, and MCF10Ca1h). Cell lines were kindly provided by The Barbara Ann Karmanos Cancer Institute (Detroit, MI, USA), except for MCF10A cells, which were purchased from The American Type Culture Collection (LGC, Teddington, UK), and MCF10DCIS.com cells from Asterand (Royston, UK). Cells were authenticated by short tandem repeat typing with the Geneprint10 Kit (Promega, Southampton, UK), and routinely tested for mycoplasma infection by use of an enzyme‐linked immunosorbent assay‐based test (MycoAlert Mycoplasma detection kit; Lonza, Basel, Switzerland). Cells were grown as described previously 30 and in supplementary materials, Supplementary materials and methods.

### Nucleic acid isolation

DNA was isolated with the DNeasy Blood and Tissue kit (Qiagen, Crawley, UK), and RNA was extracted with Trizol (Life Technologies, Paisley, UK), according to the manufacturers' instructions. Nucleic acids were quantified using the Qubit Fluorometer assay (Life Technologies), and RNA integrity was defined using a Bioanalyzer (Agilent, Santa Clara, CA, USA). All samples had an RNA integrity number of >9.

### Exome sequencing

Genomic DNA (1 µg) was subjected to DNA capture with the Human All Exome V4 XT kit (Agilent, Santa Clara, CA, USA), and sequenced on 50% of a lane on an Illumina HiSeq2500, resulting in a minimum of × 109 median depth of coverage. Paired‐end reads in FASTQ format were aligned to the reference human genome build GRCh37, by the use of Burrows–Wheeler Aligner (BWA) 31. Variants were identified with the Genome Analysis Toolkit v3 (GATK) 32, and variant annotation was performed according to GATK Best Practices recommendations with Refseq and excluding decoy sequences 33, 34. Exome DNA sequencing was also performed with the Ion Torrent AmpliSeq technology (Life Technologies), according to the manufacturer's instructions (supplementary materials, Supplementary materials and methods), with a median depth of >100 for all samples. The Torrent Suite v4.0.2 pipeline (Life Technologies) was used to align raw reads and identify variants. Calls from the GATK were overlapped with calls from the Torrent Suite v4.0.2 pipeline to identify robust mutations. Candidate somatic mutations were called on the basis of filtering of variants with minor allele frequencies of >1% according to dbSNP build 132 and with <5 supporting reads. Mutations associated with progression in any cell line from MCF10neoT onwards were subsequently annotated on the basis of calls by Strelka 35, with MCF10As as the baseline comparator and manual review with the Integrative Genomics Viewer 36 to rule out the presence of reads supporting a given mutation in the ‘negative’ cell lines. Variants were subsequently annotated with Annovar 37. Mutations were overlaid with annotated data from primary breast cancers from The Cancer Genome Atlas (TCGA) 38 and subjected to functional prediction algorithms (supplementary materials, Supplementary materials and methods). A subset of variants taken forward for functional analysis were validated in all cell lines by Sanger sequencing, as described previously 39.

### Whole genome sequencing

Libraries for whole genome sequencing were prepared with the NEBNext Ultra DNA Library Preparation kit (New England Biolabs, Hitchin, UK) from 1 µg of DNA, according to the manufacturer's protocol. Whole genome sequencing FASTQ files were aligned to the human genome (hg19) with BWA 40, and copy number variations were identified with the Patchwork 41 and GISTIC algorithms 42, as described in supplementary materials, Supplementary materials and methods. DNA was also subjected to high‐resolution microarray comparative genomic hybridization (aCGH) as described previously 43 and in supplementary materials, Supplementary materials and methods.

### Paired‐end massively parallel RNA sequencing

RNA sequencing was performed with 100 ng of ribosomal‐depleted RNA from cell lines grown on plastic [two‐dimensional (2D) and as spheroids] as described previously 44 (see supplementary materials, Supplementary materials and methods). RNA sequencing FASTQ files were aligned to the human genome (GRCh37.73) with TopHat version 2.0.8b 45. Reads mapping to two or more locations were removed from the analysis. Differential gene expression analysis was performed with DESeq2, with an adjusted *p*‐value cut‐off of ≤0.01 46. Gene expression of the preinvasive cells (MCF10A, MCF10AT1, and MCF10neoT) was compared with that of the invasive cells (MCF10Ca1a, MCF10Ca1d, and MCF10Ca1h). MCF10DCIS.com cells were omitted from this analysis, given that they form preinvasive lesions *in vivo* that spontaneously become invasive 47. Fusion genes were identified with Chimerascan 48 and deFuse 49 algorithms. Pathway enrichment was performed with ConsensusPathDB 50.

## Reverse transcription‐quantitative PCR (RT‐qPCR) and Sanger sequencing validation

Reverse transcription was performed with Superscript III (Invitrogen), with 500 ng of RNA per reaction, as described previously 51 and in supplementary materials, Supplementary materials and methods. Sequences were visualized by the use of 4Peaks (http://nucleobytes.com/4peaks/). In‐frame fusion genes were quantified in the cell line series with RT‐qPCR, and the abundance of the fusion transcript relative to β‐actin mRNA (*ACTB*) was calculated with the delta‐delta CT method. Primer sequences are listed in supplementary materials, Table S1.

### Small interfering RNA (siRNA) screen

Genes were chosen to be screened with siGENOME smartpool siRNA (GE Healthcare, Little Chalfont, UK) targeting wild‐type genes in 96‐well spheroid assays, based on the presence of either: (1) recurrent amplifications and homozygous deletions, or (2) non‐synonymous coding mutations in the progression series. Alterations were chosen that were also present in primary tumours assessed in METABRIC 52 and aCGH studies 53, 54, 55 for copy number alterations, and from TCGA and other published studies at a frequency >0.5% for somatic mutations 56, 57, 58, 59, 60. For amplifications and homozygous deletions, those that are either known drivers or predicted drivers (for amplifications) as assessed from a significant correlation of amplification with gene expression 55 were selected. For mutations, those that are known to be drivers or predicted to be drivers from the prediction algorithm FATHMM 61 were triaged for functional assessment.

### Three‐dimensional spheroid cultures

Five thousand cancer cells per well of a 96‐well low‐attachment plate (Corning, Amsterdam, The Netherlands) were reverse‐transfected with 37.5 nm siGENOME smartpool siRNA (GE Healthcare) or siControls [positive (ubiquitin B) and negative (pool 1)] by the use of Lullaby reagent (Oz Biosciences, Marseille, France) in 180 µl of cold culture medium, as described previously 62. Spheroid area was calculated with Celigo S (Nexcelom, Lawrence, MA, USA), and viability was measured with the CellTiter‐Glo assay (Promega). Relative growth was calculated relative to siControl. Hits were scored as >1.2 for increased spheroid growth and as <0.8 for reduced cell growth (see supplementary materials, Supplementary materials and methods).

### Transfections of mammalian cells on plastic

Two thousand five hundred cancer cells per well of a 96‐well plate (Sigma‐Aldrich, Dorset, UK) were reverse‐transfected with 37.5 nm siGENOME siRNA by the use of Lullaby reagent (Oz Biosciences), as described previously 54 and in supplementary materials, Supplementary materials and methods.

### Immunohistochemistry of spheroid cultures

Spheroids were grown for 7 days, and fixed in 3.8% formaldehyde for 30 min, washed with phosphate‐buffered saline three times, and stored at 4 °C. Spheroids were then pooled, dehydrated, embedded in paraffin, and sectioned. The spheroid sections were then deparaffinized with xylene, rehydrated, microwaved, and incubated overnight with primary antibodies against Ki67, TP53, and pAKT (see supplementary materials, Supplementary materials and methods).

### Statistical analyses

*p*‐Values of <0.05 (heteroscedastic, two‐sided) were considered to be statistically significant for comparisons of the siRNA screen.

### Data availability

Raw whole genome, exome and RNA sequencing data have been deposited in the NCBI Sequence Read Archive under the accession number PRJNA308098.

## Results

### Genomic alterations associated with breast cancer progression

To better understand the role of genomic and transcriptomic alterations in breast cancer progression, we performed whole exome, low‐depth whole genome and RNA sequencing of the MCF10 progression series (Figure 1A) to comprehensively define the repertoire of mutations, copy number alterations, expressed fusion genes and transcriptional alterations. Whole exome sequencing was performed with both capture and amplicon‐based sequencing at an average depth of × 100. This identified 7275 coding non‐synonymous variations [single‐nucleotide variants (SNVs)] in MCF10A, 7327 in MCF10neoT, 7336 in MCF10AT1, 7364 in MCF10DCIS.com, 7354 in MCF10Ca1a, 7351 in MCF10Ca1d, and 7358 in MCF10Ca1h. Taking MCF10A cells as the baseline, we identified mutations in 196 genes that were acquired in the malignant cell lines (i.e. not present in MCF10A non‐malignant cells; supplementary materials, Table S2), including 64 genes that also occur in TCGA and other published DNA sequencing studies 56, 57, 58, 59, 60 (Figure 1B). These included a *PIK3CA* hotspot mutation (H1047R) acquired in MCF10DCIS.com cells and maintained in the invasive cell lines MCF10Ca1a and MCF10Ca1h (in agreement with previous reports 63), and novel convergent mutations in *TP53* in MCF10Ca1a and MCF10Ca1h cells. We next defined the presence of relevant breast cancer predicted driver gene mutations that were acquired in the malignant cells by annotating the variants with a combination of functional prediction algorithms (see Materials and methods) and the specific cancer driver prediction algorithm FATHMM 61. This analysis revealed 53 mutations that were predicted to disrupt protein function, and seven predicted cancer driver mutations. These encompassed four predicted cancer driver mutations that were acquired during transformation of non‐malignant MCF10A cells to malignant DCIS.com cells (*HRAS*, *EPHA7*, *MAP3K12*, and *PCSK5*), and three that were acquired during transformation of MCF10DCIS.com cells to invasive cells (MCF10Ca1a and MCF10Ca1h) (*PTPRD*, *TP53*, and *VSP13A*) (Figure 1E; supplementary material, Figures S1 and S2). Furthermore, 57% of all variants were expressed at the RNA level (supplementary material, Table S2).

### Somatic copy number alterations associated with progression

We used low‐depth [on average, ×7 (range of coverage, ×6–9] whole genome sequencing to characterise the repertoire of copy number alterations of cell lines within the progression series. Consistent with previous observations 24, 63, MCF10A cells had high‐level gains of 1q, gains of 5q, 8q, 19q, and 20q, and homozygous deletion of 9p encompassing *CDKN2A*/*B* (supplementary material, Figure S3). Indeed, the other cell lines were comparable, with high‐level focal amplification of 8q24.21, encompassing *MYC*, 10q22.1–q22.2, and 17p11.2 (Figure 1D; supplementary material, Table S3). They did not have gain of 1q, and were therefore probably derived from a clone of the parental line that had normal 1q (Figure 1C). Interestingly, MCF10DCIS.com cells had both the gain of 1q seen in the parental MCF10A cells and the three focal high‐level amplifications. A number of homozygous deletions were acquired during progression, including 8p23.1 (MCF10DCIS.com, MCF10Ca1a, and MCF10Ca1d), 12p13.2 (MCF10AT1, MCF10DCIS.com, and MCF10Ca1a) and 22q12.2 (MCF10neoT, MCF10AT1, MCF10DCIS.com, MCF10Ca1a, and MCF10Ca1d) (Figure 1C; supplementary material, Figures S3 and S4 and Table S3). In addition, acquisition of a focal intragenic homozygous deletion of *RUNX1* (MCF10DCIS.com, MCF10Ca1a, and MCF10Ca1d) was identified (in agreement with previous reports 63).

### Fusion gene transcripts associated with progression

**Figure 2:**
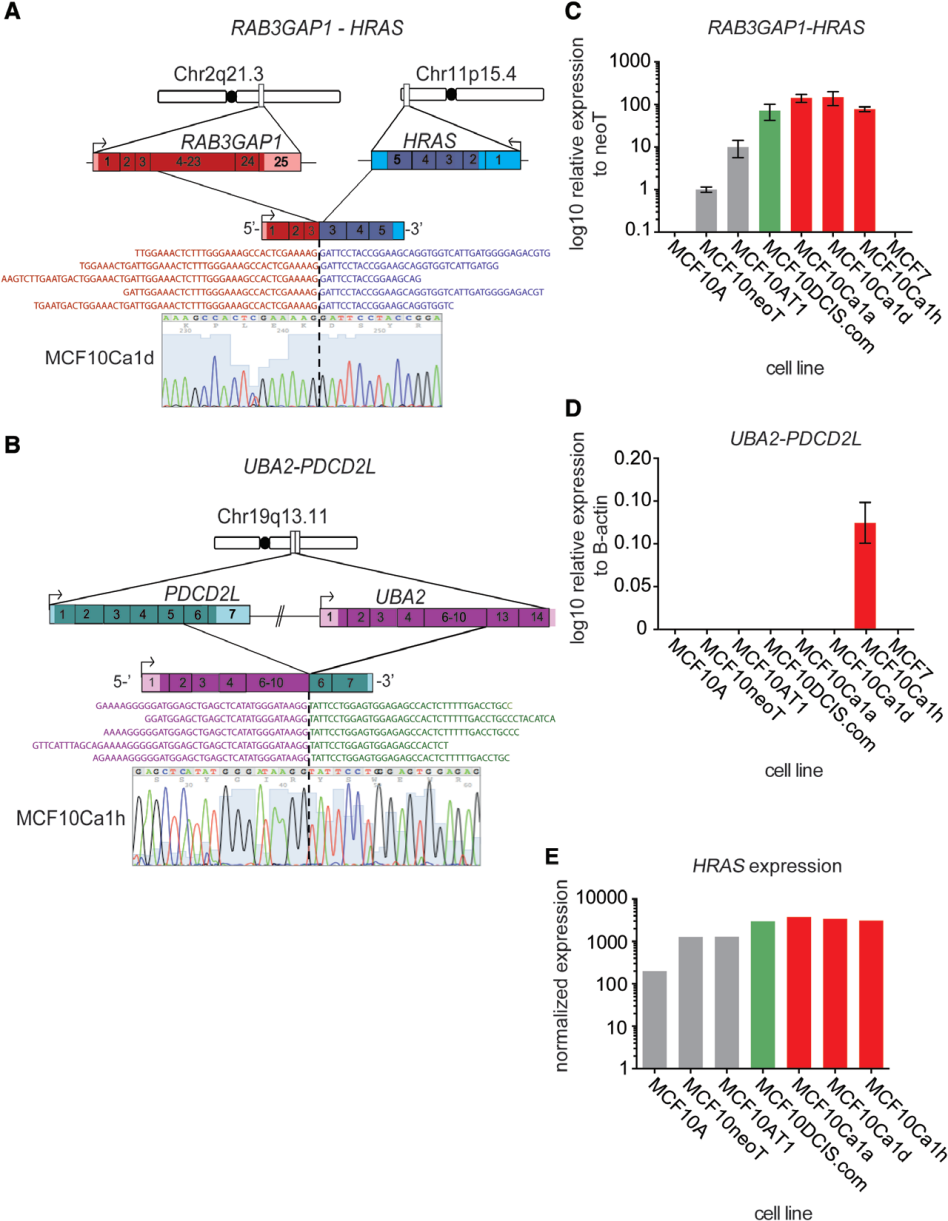
Identification of expressed fusion genes in the MCF10 progression series. (A) Cartoon representation of genomic location, orientation and architecture of the expressed RAB3GAP1–HRAS fusion gene. Representative RNA sequencing reads spanning the fusion are also displayed. The RT‐qPCR product was Sanger‐sequenced; confirming the fusion junction, and a representative chromatogram from MCF10Ca1d cells is shown. (B) Cartoon representation of genomic location, orientation and architecture of the expressed UBA2–PDCD2L fusion gene. Representative RNA sequencing reads spanning the fusion are also displayed. The RT‐qPCR product was Sanger‐sequenced, confirming the fusion junction, and a representative chromatogram from MCF10Ca1h cells is shown. (C) Bar plot showing relative expression of the RAB3GAP1–HRAS fusion gene in the MCF10 progression series detected by RT‐qPCR. (D) Bar plot showing relative expression of the UBA2–PDCD2L fusion gene in the MCF10 progression series detected by RT‐qPCR (E) Bar plot of normalized reads of HRAS in the MCF10 progression series from RNA sequencing.

Previous studies have reported that breast cancers can show extensive large‐scale genomic rearrangements 64, and have documented the presence of expressed fusion genes that drive the malignant phenotype of the cells and present therapeutic opportunities 43, 65. RNA sequencing analysis of the MCF10 progression series identified expressed fusion genes in MCF10DCIS.com (*n* = 1), MCF10Ca1d (*n* = 1) and MCF10Ca1h (*n* = 2) that were identified by both deFUSE 49 and Chimerascan 48 fusion gene detection algorithms (supplementary material, Table S4). These included two fusion transcripts predicted to result in novel functional proteins (i.e. in‐frame) that were not present in MCF10A cells, namely an interchromosomal fusion involving *RAB3GAP1* and *HRAS*, detected in MCF10DCIS.com and MCF10Ca1d cells, and an intrachromosomal fusion on chromosome 19q involving *UBA2* and *PDCD2L* in MCF10Ca1h cells (Figure 2A, B). Validation of the fusion transcripts with RT‐qPCR and Sanger sequencing demonstrated that the in‐frame *RAB3GAP1–HRAS* fusion was present in all cell lines that had been subjected to HRAS transformation (i.e. from MCF10AT1 onwards), whereas *UBA2–PDCD2L* was only seen in MCF10Ca1h cells (Figure 2C, D; supplementary material, Table S4). Neither fusion was detected in the control cell line MCF7. Furthermore, the levels of *RAB3GAP1–HRAS* transcript expression increased from MCF10neoT cells across the progression series, with MCF10Ca1d cells showing the highest expression. This observation mirrored the *HRAS* gene expression levels detected in the RNA sequencing data (Figure 2E), suggesting that the differences in HRAS expression may be attributable to the presence of the fusion gene. Interestingly, the reciprocal fusion *HRAS–RAB3GAP1* was detected in MCF10DCIS.com cells; however, this was not predicted to result in a functional protein. Mining of the TCGA Fusion gene Data Portal (http://54.84.12.177/PanCanFusV2/) 66 and other published fusion datasets in breast cancer 43, 64, 65 identified two additional in‐frame fusion genes involving *RAP3GAP1* in breast cancer (*RAB3GAP1–ACMSD* and *RAB3GAP1–MAP4K3*). An in‐frame *HRAS* fusion gene was identified in a head and neck primary tumour (*RNH1–HRAS*) that leads to increased levels of HRAS expression 66; however, no additional *HRAS* fusion genes were detected in primary breast cancers. An out‐of‐frame *UBA2–PDCD2L* fusion was detected in a primary ovarian cancer, but none was observed in breast cancer.

### Pathway alterations associated with breast cancer progression

**Figure 3:**
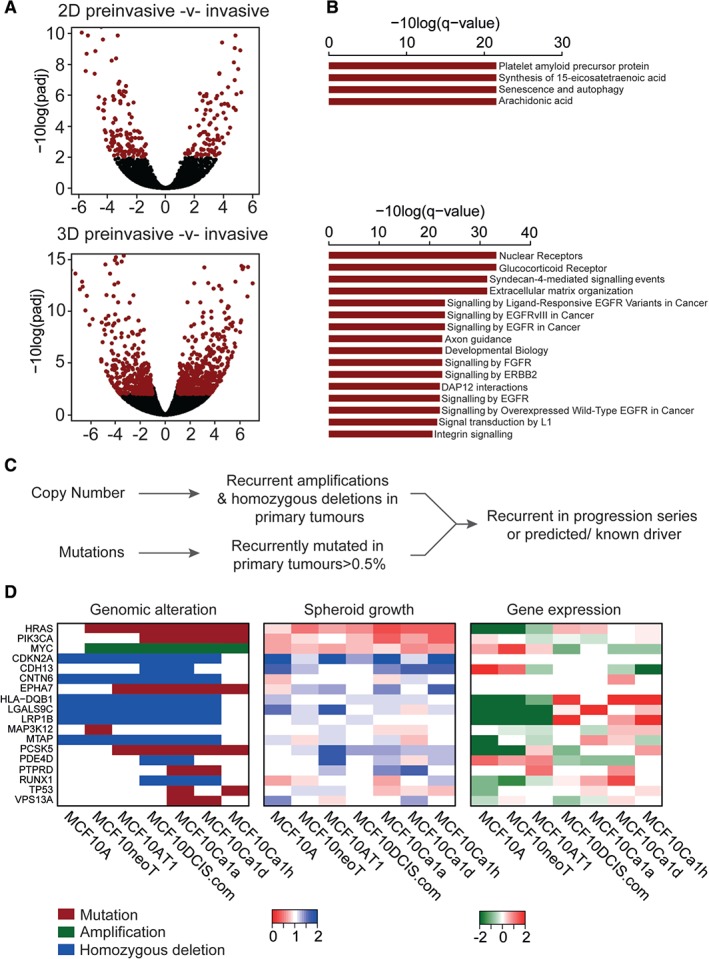
Evaluation of pathways and driver alterations in spheroid cultures. (A) Volcano plots showing the differentially expressed transcripts between preinvasive and invasive cells from the MCF10 progression series cultured under both 2D and 3D conditions. Red: FDR p‐values of <0.01. (B) Bar plot showing the significantly over‐represented pathways (ConsensusDb q‐value of <0.01) from (A). (C) Schematic of gene selection for the siRNA screen. (D) Matched heatmaps of genomic status (mutation, amplification, and homozygous deletion); results of spheroid growth after siRNA‐mediated silencing and gene expression. Relative spheroid growth was measured according to the survival fraction of treated cells relative to siControl. Hits were triaged as a relative survival fraction as compared with non‐targeting control of <0.8 or >1.2. Gene expression is the log_2_ median centred normalized reads from the RNA sequencing data.

We next sought to assess the differences in gene expression during progression from preinvasive to invasive disease. Differential gene expression of preinvasive cell lines (MCF10A, MCF10AT1, and MCF10NeoT) and invasive cell lines (MCF10Ca1a, MCF10Ca1d, and MCF10Ca1h) identified 236 significantly differentially expressed genes [false discovery rate (FDR) *p*‐value of <0.01; DEseq2] (Figure 3A; supplementary material, Table S5). These genes were enriched in pathways involved in platelet amyloid precursor protein processing, senescence, autophagy, and arachidonic acid metabolism (Figure 3B; supplementary material, Table S6). Previous studies have demonstrated that the MCF10 progression series behave differently when grown in 3D culture, and provide a useful model for studying driver alterations associated with oncogenic transformation 67, 68 and disease progression 69, 70. Indeed, cell lines from the progression series formed spheroids, and showed good growth kinetics and positive histological staining of the proproliferative markers Ki67 and phospho‐AKT (supplementary material, Figure S5). To further evaluate functional pathways that may be dysregulated in breast cancer progression, in cells grown in more *in vivo*‐like conditions 62, we performed RNA sequencing of the series of cell lines grown as 3D spheroids. This analysis identified 1022 genes that were differentially expressed between preinvasive and invasive cell lines (supplementary material, Table S5). Functional annotation of these genes identified significant over‐representation of pathways involved in nuclear receptor signalling, epidermal growth factor receptor (EGFR) signalling, ErbB receptor signalling, fibroblast growth factor receptor (FGFR) signalling, signal transduction, integrin signalling, and extracellular matrix organization (Figure 3A, B; supplementary material, Table S6). These findings indicate that more cancer‐relevant pathways are active when cells are grown in a 3D environment, possibly reflecting the way in which the cells were selected for *in vivo* when they were generated 19.

### Functional characterisation of driver alterations upon progression

**Figure 4:**
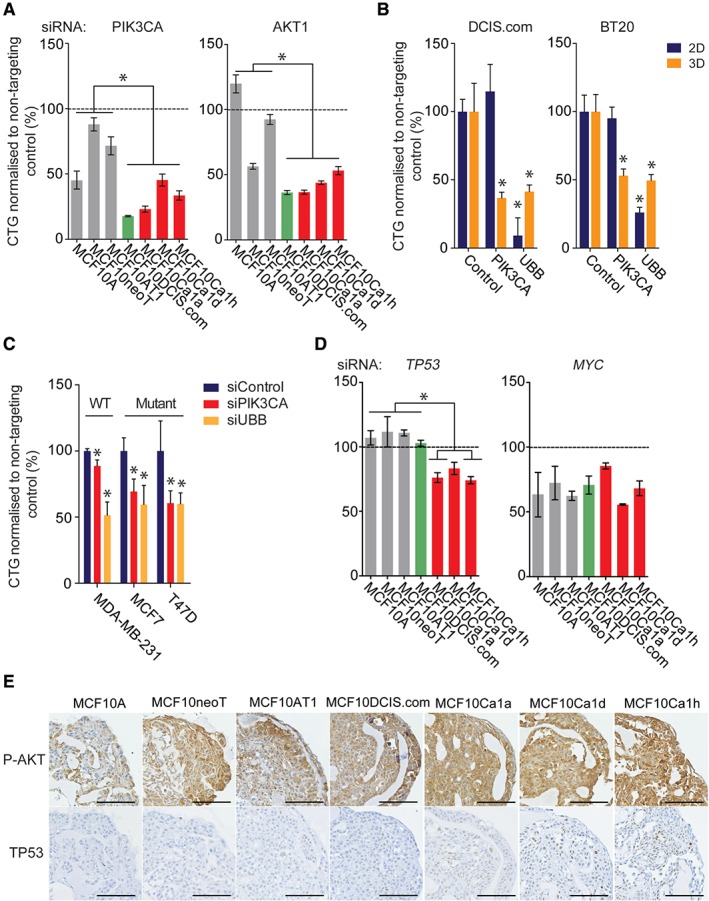
Functional validation of dependency on driver events in the MCF10 progression series. (A) Progression series cell lines were reverse‐transfected with siRNAs against PIK3CA and AKT1, and with a non‐targeting control. Spheroids were formed after 24 h in low‐attachment plates, and the medium was topped up every 3 days. After 7 days, spheroid viability was determined with Cell Titre Glo. (B) DCIS.com and BT20 cell lines were reverse‐transfected with siRNAs targeting PIK3CA and UBB, and with a non‐targeting control, under both 2D and 3D conditions. The medium was topped up every 3 days. Viability was determined with Cell Titre Glo. Statistical comparisons were performed with Student's t‐test (*p ≤ 0.05). (C) MDA‐MB‐231, MCF7 and T47D cell lines were reverse‐transfected with siRNAs targeting PIK3CA and UBB, and with a non‐targeting control, under 3D conditions. The medium was topped up every 3 days, and spheroid viability was determined with Cell Titre Glo. Statistical comparisons were performed with Student's t‐test (*p ≤ 0.05). (D) Progression series cell lines were reverse‐transfected with siRNAs against TP53 and MYC, and with a non‐targeting control. Spheroids were formed after 24 h in low‐attachment plates, and the medium was topped up every 3 days. After 7 days, spheroid viability was determined with Cell Titre Glo. (E) The progression series was grown for 7 days. The medium was topped up every 3 days. After 7 days, spheroids were fixed with formaldehyde, embedded, sectioned, and stained for phospho‐AKT (P‐AKT) (473) and total TP53. Representative images are shown. Scale bar: 100 µm.

Given our observations that, when grown in spheroid cultures, the MCF10 cell line series show enrichment of cancer‐relevant pathways associated with progression to invasive disease, we sought to functionally test which genomic alterations (amplifications, homozygous deletions, and mutations) that are also seen in primary breast cancers (Figure 3C; see Materials and methods) would be driving the growth of these cells. Cell lines were optimized for siRNA‐mediated gene depletion, whereby ablation of the genes encoding the tumour suppressor phosphatase and tensin homolog and ubiquitin B resulted in increased and decreased spheroid growth relative to control siRNA, respectively (supplementary material, Figure S5). A siRNA‐based screen of 18 genes identified three that constitute potential driver events, namely *PIK3CA* (*p* = 0.0485, *t*‐test), *HRAS*, and *TP53* (*p* < 0.0001, *t*‐test), and that, when silenced, decreased spheroid growth and were associated with genomic status (Figure 3D; supplementary material, Table S7). Deconvolution of the siRNA oligonucleotide pools showed that all of these genes were oncogenic drivers, resulting in decreased spheroid growth when silenced (supplementary material, Figure S6). These included *PIK3CA*, whereby cells with an H1047R activating mutation showed selective dependency on *PIK3CA* silencing (Figure 4A). In addition, *PIK3CA* mutant cells were also selectively dependent on *AKT1* silencing (*p* = 0.046, *t*‐test), perhaps reflective of the subsequent increased AKT1 activation, (Figure 4A; supplementary material, Figures S5 and S6); however, this appeared to be an effect specific to cells grown as spheroids, and was not observed in traditional 2D culture (Figure 4B; supplementary material, Table S7). Furthermore, the oestrogen receptor‐negative breast cancer cell line BT20, which harbours an H1047R *PIK3CA* mutation, showed a similar effect (Figure 4B). Moreover, breast cancer cell line spheroids showed dependency on PIK3CA according to their *PIK3CA* status, with mutant MCF7 and T47D cells (harbouring E575K and H1047R *PIK3CA* mutations, respectively) being sensitive to *PIK3CA* silencing, and MDA‐MB‐231 cells (wild type) showing no change in viability after *PIK3CA* silencing. It is tempting to posit that this is due to the maintenance of AKT activity under unfavourable conditions imparted by the spheroid architecture, as spatial AKT activity was observed in the preinvasive cell lines, whereas stable and high phospho‐AKT staining was observed in the invasive cell line spheroids that harboured the activating mutation in *PIK3CA* (Figure 4E).

Interestingly we observed two independent SNVs in MCF10Ca1a and MCF10Ca1h cells in the DNA‐binding domain of TP53, suggestive of convergent evolution. *TP53* silencing in the progression series significantly correlated with smaller spheroid size in mutant cells, suggesting that these mutations act in an oncogenic manner (*p* = 0.0051, *t*‐test). Moreover, there was a significant dependency on TP53 associated with increased progression (preinvasive versus invasive cells; *p* = 0.0021, *t*‐test), regardless of mutation status, that appeared to correlate with increased nuclear accumulation of TP53 protein in these cells (Figure 4D, E). It is of note that all cell lines showed sensitivity to *MYC* silencing, suggesting that cells are dependent on *MYC* transcriptional activity independently of amplification status (Figure 4D).

**Figure 5:**
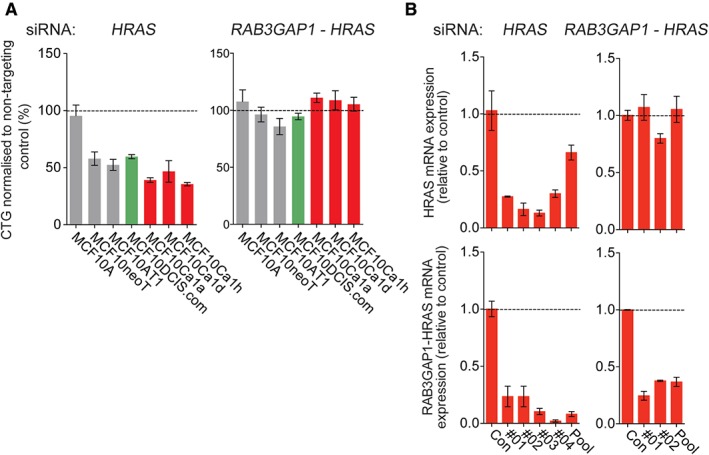
Functional validation of dependency on TP53 and HRAS in the MCF10 progression series. (A) Progression series cell lines were reverse‐transfected with siRNAs against HRAS and RAB3GAP1–HRAS, and with a non‐targeting control. Spheroids were formed after 24 h in low‐attachment plates, and the medium was topped up every 3 days. Spheroid viability was determined with Cell Titer Glo. (B) MCF10Ca1a cells were reverse‐transfected with single and pooled siRNAs targeting HRAS and RAB3GAP1–HRAS, and with a non‐targeting control, for 72 h. HRAS expression and RAB3GAP1–HRAS expression were determined with RT‐qPCR. Expression was normalized to loading controls B2M and β‐actin (ACTB).

Given that MCF10A cells underwent *HRAS* transformation to produce subsequent cell lines, we tested whether cells would still be dependent on oncogenic RAS signalling for their survival further along the course of progression for cell survival. Indeed, silencing of *HRAS* reduced spheroid growth of all cells subsequent to MCF10A (Figure 5A); however, this association appeared to be significantly correlated with expression of the *RAP3GAP1–HRAS* fusion gene (*r* = –0.7857, *p* = 0.0480, Spearman rank correlation) rather than on total *HRAS* expression (*r* = –0.5714, *p* = 0.2, Spearman rank correlation), and, in a similar manner to *PIK3CA* and *AKT1*, seemed to be more specific to cells grown as spheroids (supplementary material, Figure S5). Specific silencing of the *RAB3GAP1–HRAS* fusion gene, however, had no effect on spheroid growth (Figure 5A, B), perhaps indicative of the subclonal nature (as evidenced by the low percentage of the transcript involved in the fusion, i.e. isoform fraction) of the cells in which the fusion was detected with RNA sequencing (supplementary material, Table S4).

## Discussion

Here, we have performed a comprehensive analysis of both the genomes (at base pair resolution) and transcriptomes of the MCF10 cell line series that represent different stages of breast cancer progression when grown *in vivo*, and have demonstrated that these cell lines harbour relevant driver alterations seen in primary breast cancers, and represent a good model for studying breast cancer progression with *in vitro* spheroid models.

Overall, the patterns of genomic copy number alterations are similar between the cell lines; however, there are key differences, suggestive of subclonal selection from the parental MCF10A cells. In particular, at base pair resolution, the number of mutations varied across cell lines, with a number of key driver mutations being selected for at different stages of progression. These included a *PIK3CA* hotspot mutation in MCF10DCIS.com cells that leads to increased AKT signalling, and *TP53* mutations in the more aggressive invasive cell lines MCF10Ca1a and MCF10Ca1h 71, as shown by increased nuclear accumulation of TP53 protein in these cells. We identified a number of mutations that were clonally selected through progression for which the allele fraction differed in the different cell lines. It may be the case that such alterations are merely passengers (i.e. do not confer a selective advantage to the cells); however, they may provide a growth advantage at different stages of progression, which is in agreement with recent studies on triple‐negative breast cancers, where genuine driver alterations have been shown to be subclonal and heterogeneously distributed 59. At the copy number level, focal high‐level *MYC* amplification was acquired in the malignant cell lines, as previously reported 63; however, it was not seen in MCF10A cells, which harboured gain of the entire arm of 8q. This finding is in agreement with other reports showing gain of *MYC* to be an initiating event in this cell line panel, rather than focal amplification 72. Indeed, *MYC* amplification has been associated with a poor prognosis 73, 74, and is often acquired in metastatic disease 75, 76. However, all cells in the progression series appeared to be sensitive to *MYC* silencing. Although the majority of the cell lines appeared to be derived from a clone lacking 1q gain, the presence of 1q gain in MCF10DCIS.com cells may alternatively be a result of isochromosome 1q being lost in culture.

Consistent with previous observations that members of the MCF10 progression series behave differently when grown in 3D culture 69, 70, we found a number of distinct differentially regulated pathways associated with progression when cells were grown in spheroids as compared with traditional 2D culture, perhaps reflecting the nature of the nutrient and oxygen gradients in these models 62. Indeed, functional assessment of recurrent alterations identified oncogenic dependencies that were only observed when assessed in spheroid models, including a selective dependency on PIK3CA signalling in cells harbouring an H1047R mutation that was corroborated in additional breast cancer cells harbouring the H1047R mutation. Indeed, the H1047R mutation has been shown to promote metabolic adaption by increasing *de novo* lipogenesis 77, a feature observed in aggressive cancers.

Through exome sequencing, we identified independent non‐synonymous coding mutations in *TP53* in the MCF10Ca1a and MCF10Ca1h cell lines. Consistent with observations that *TP53* mutations can be late events in breast cancer progression 59 and are associated with a poor prognosis 78, it has been shown that these sublines can spontaneously metastasise when grown *in vivo* 19. Interestingly, in all the invasive cells (MCF10Ca1a‐1 h), increased nuclear TP53 protein accumulation was observed, which correlated with sensitivity to *TP53* silencing, suggesting that TP53 dysregulation is associated with increased cellular invasiveness and an oncogenic dependency in this model.

In addition to the identification of mutations associated with progression, we also identified two in‐frame fusion genes in the cell line series, included a *HRAS* fusion that was acquired in MCF10neoT cells, which were transformed by oncogenic HRAS, and was maintained in all subsequent cell lines. This fusion gene joins the promoter of *RAB3GAP1*, which encodes a GTPase‐activating protein, to exon 3 of *HRAS*. Although it is interesting to speculate that this fusion gene would also lead to selective HRAS dependency, given an observed correlation of HRAS oncogenic dependency and expression of the fusion gene, we observed no effect on spheroid growth with selective inhibition of the fusion gene. This may be because the fraction of fusion transcript as compared with that wild‐type transcript represents ∼1% of all *HRAS* reads. As no additional *HRAS* fusion genes were observed on analysis of published RNA sequencing data, this fusion most likely represents a consequence of HRAS v12 transformation.

Our study, although comprehensive, is not without limitations. *HRAS* mutation is not a common genetic alteration in human breast cancer, so the cell line series model might not accurately reflect the tumorigenic process in human breast cancers. This is exemplified by the identification of a fusion gene involving *HRAS* in the model and the lack of such fusion genes in primary breast cancer. Although exome sequencing identified the acquisition of mutations in the malignant cells, we cannot rule out the possibility that these are present at very low subclonal populations in the parental MCF10A cells, given that we did not perform high‐depth targeted resequencing. Nevertheless, through exome sequencing with an average depth of × 100, we observed clonal selection of alterations across different cell lines, suggesting that MCF10A cells are oligoclonal. Although our copy number data support this, the low depth of coverage may also limit the detection of subclonal events. Our triage of genomic alterations for functional assessment mainly identified mutational events and homozygous deletions rather than amplifications that were representative of primary breast cancers. This may be due to our triage strategy; however, it is known that, in general, triple‐negative breast cancers lack many recurrent amplification events 59. Moreover, genomic alterations tested that did not cause a difference in spheroid growth may score in additional assays, and may warrant further testing. In addition, functional assessment of differentially expressed genes may provide further insights into the drivers of progression in this model.

In conclusion, comprehensive characterisation of the MCF10 isogenic progression series has identified a number of driver alterations that are associated with progression from preinvasive to invasive cellular phenotypes that model the genomic alterations seen in primary breast cancer. Moreover, more accurate modelling of the *in vivo* tumour environment with 3D culture methods allows the validation of founder (HRAS transformation) and acquired (*PIK3CA* and *TP53* mutations) events that would not have been appreciated with traditional techniques. The MCF10 progression series therefore represents a good model with which to dissect potential biomarkers and evaluate therapeutic targets involved in the progression of breast cancer.

## Author contributions statement

The authors contributed in the following way: RN: conceived the study; KB, GF: provided materials; BP, PTW, SV, FD, RN carried out the experiments: SLM, JC, HB, AG: performed the bioinformatics analysis; SLM, BP, PH, CJL, RN: discussed and interpreted the results; SLM, BP, CJL, RN: wrote the first draft. All authors read and approved the final manuscript.

**SUPPLEMENTARY MATERIAL ONLINE**

**Supplementary materials and methods**

**Supplementary figure legends**

**Figure S1.** Variant allele frequency plots of acquired mutations.

**Figure S2.** IGV screenshots of acquired mutations in the MCF10 progression series.

**Figure S3.** Copy number alterations in the MCF10 progression series.

**Figure S4.** Focal amplifications in the MCF10 progression series.

**Figure S5.** RNAi modulation of the isogenic MCF10 model in cancer cell line spheroids.

**Figure S6.** Oligo deconvolution in MCF10Ca1a cells in spheroids and on plastic.

**Table S1.** Details of primer sequences, siRNA sequences and qPCR assays.

**Table S2.** Mutations identified in the MCF10 progression series.

**Table S3.** Gains and losses and focal amplifications and deletions from GISTIC.

**Table S4.** Expressed fusion genes identified from RNA‐sequencing of cell lines grown as spheroids identified by both DeFuse and Chimerascan algorithms.

**Table S5.** Differentially expressed genes between pre‐invasive and invasive cells grown on plastic and as spheroids.

**Table S6.** Pathway analysis of differentially expressed genes between pre‐invasive and invasive cell lines grown on plastic and as spheroids.

**Table S7.** Summary of siRNA screen of cells grown as spheroids and on plastic.

## Supporting information


**Supplementary materials and methods**

**Supplementary figure legends**

**Figure S1: Variant allele frequency plots of acquired mutations** Variant allele frequency (VAF) plots of predicted driver mutations acquired in malignant cells, depicting the cell line (x‐axis) and VAF (y‐axis). The size of the dots corresponds to the depth of coverage.

**Figure S2: IGV screenshots of acquired mutations in the MCF10 progression series** Screenshots from Integrative Genomics Viewer (IGV) of driver genes acquired from in malignant cells, showing the presence or absence of mutant reads in the cell lines. Reads are sorted according to mutant reads.

**Figure S3: Copy number alterations in the MCF10 progression series** Genome plots showing normalised coverage of reads (y‐axis) from whole genome sequencing are plotted according to the genomic position in the genome (x‐axis).

**Figure S4: Focal amplifications in the MCF10 progression series** (A) Chromosome 10 and (B) Chromosome 17 plots showing focal amplifications acquired during progression. Normalised coverage (y‐axis) is plotted according to genomic position (x‐axis)

**Figure S5: RNAi modulation of the isogenic MCF10 model in cancer cell line spheroids** (A) Micrograph images of MCF10DCIS.com cells subsequent to transfection of siRNA. Images were taken at day 7. (B) Heatmap of spheroid growth after siRNA mediated silencing and gene expression on plastic (2D). Relative spheroid growth is measured by the survival fraction of treated cells relative to siControl. (C) Micrograph images of spheroids at day 1, 4 and 7 of the MCF10 progression series, demonstrating good growth kinetics. (D) Images of spheroids stained with H&E and antibodies against Ki67, phosphor‐AKT, and TP53.

**Figure S6: Oligo deconvolution in MCF10Ca1a cells in spheroids and on plastic**. (A) Barplots showing relative mRNA levels after gene silencing. Statistically significant alterations in mRNA expression were calculated using Student's *t* test (*p ≤ 0.05) (B) Barplots of relative cell growth in 2D and as 3D spheroids with individual oligos. Relative spheroid growth is measured by the survival fraction of treated cells relative to siControl. Statistical comparisons were performed using Student's *t*‐test (*p ≤ 0.05)

**Table S1** Details of primer sequences, siRNA sequences and RT‐qPCR assays.

**Table S2** Mutations identified in the MCF10 progression series.

**Table S3** Gains and losses and focal amplifications and deletions from GISTIC.

**Table S4** Expressed fusion genes identified from RNA‐sequencing of cell lines grown as spheroids identified by both DeFuse and Chimerascan algorithms.

**Table S5** Differentially expressed genes between pre‐invasive and invasive cells grown on plastic and as spheroids.

**Table S6** Pathway analysis of differentially expressed genes between pre‐invasive and invasive cell lines grown on plastic and as spheroids.

**Table S7** Summary of siRNA screen of cells grown as spheroids and on plastic.
