# Supplementary material for: Three‐dimensional modelling identifies novel genetic dependencies associated with breast cancer progression in the isogenic MCF10 model
Source: J Pathol. 2016 Oct 19;240(3):315–28. doi: 10.1002/path.4778 (PMC5082563; doi:10.1002/path.4778)
Supplement: Supplementary file 1 — Supplementary materials and methods [file PATH-240-315-s003.docx]

**Supplementary materials and methods**

**Cell lines**

MCF10CA1a, MCF10CA1d, MCF10CA1h, and MCF10DCIS.com cells were cultured in minimal DMEM/F12 medium supplemented with 2 mM L-glutamine, 15mM HEPES, 3.151 g/L-glucose, 5% horse serum, and 0.028M sodium bicarbonate [[25](#_ENREF_25)]. MCF10A, MCF10AT1 and MCF10NeoT cells were grown in complete medium composed of the whole minimal DMEM medium components supplemented with 100 ng/ml cholera toxin, 20 ng/ml epidermal growth factor, 10 μg/ml insulin, and 0.5 μg/ml hydrocortisone as described [[67](#_ENREF_67)]. Cells were incubated with 5% CO2 at 37 °C.

**Exome sequencing**

Genomic DNA (1 μg) was fragmented to 200 bp using a Covaris E Series instrument (Covaris, MA, USA) and the resultant library subjected to DNA capture, using the Human All Exome V4 XT kit (Agilent, CA, USA), according to the manufacturer’s instructions. Illumina paired-end libraries were prepared from the captured target regions and quantified using a Bioanalyzer DNA chip (Agilent), followed by sequencing on a HiSeq2000 platform (Illumina, San Diego, CA, USA), using v3 chemistry acquiring 2 × 76 bp reads. Each library was sequenced on 50% of a lane to result in a minimum of 109X median depth of coverage. Casava software (v. 1.8, Illumina) was used to make base calls. Sequences were output in FASTQ format. Reads failing the Illumina chastity filter were removed before further analysis. Exome sequencing FASTQ files were aligned to the human genome (hg19) using Burrows-Wheeler Aligner (BWA) [[40](#_ENREF_40)] and initial quality control was carried out. Variants were identified using the Genome Analysis Toolkit v3 (GATK) [[32](#_ENREF_32)] base quality score recalibration, indel realignment, duplicate removal, and performed SNP and INDEL discovery and genotyping across all samples simultaneously using standard hard filtering parameters or variant quality score recalibration according to GATK Best Practices recommendations [[33](#_ENREF_33),[34](#_ENREF_34)].

Exome DNA sequencing was also performed using the Ion Torrent AmpliSeq technology (Life Technologies, Paisley UK), according to the manufacturer’s instructions. Briefly, 1ug of DNA was amplified using pooled primer sets and quantified using Ion Library Quantitation Kit (Life Technologies). According to standard protocols. Samples were sequenced on the Ion Torrent with the Ion PI Chip v2, 2 samples per chip. 30 -33 million reads were obtained per sample with median depth >100 for all samples. The Torrent Suite v4.0.2 pipeline (Life Technologies) was used to align raw reads and identify variants. Calls from the GATK where overlapped with calls from The Torrent Suite v4.0.2 pipeline to identify robust mutations. Somatic mutations were called based on filtering of variants with minor allele frequencies >1% according to dbSNP build 132 and with <5 supporting reads. Mutations associated with progression in any cell line from MCF10neoT onwards were subsequently annotated based on calls by Strelka [[35](#_ENREF_35)] using MCF10As as the baseline and manual review using the Integrative Genomics Viewer [[36](#_ENREF_36)]. Variants were annotated using Annovar [[37](#_ENREF_37)] using build 37 of the genome. Mutations were overlaid with annotated data from primary breast cancers from The Cancer Genome Atlas (TCGA) [[38](#_ENREF_38)]. Mutations were subjected to annotation with functional prediction algorithms including the cancer prediction algorithm FATHMM [[61](#_ENREF_61)], and also SIFT, PolyPhen, Mutation Taster and Mutation Assessor as implemented through Annovar [[37](#_ENREF_37)]. A subset of variants taken forward for functional analysis were validated in all cell lines using Sanger Sequencing as described [[39](#_ENREF_39)].

**Whole genome sequencing**

One microgram of genomic DNA was fragmented to 500 bp using a Covaris E Series instrument (Covaris Inc.). Libraries for whole genome sequencing were prepared following the NEBNext Ultra DNA Library Preparation kit (New England Biolabs) according to the manufacturer’s protocol. Size selection for 600-800bp fragments was used, together with 2 cycles of PCR amplification. NEBNext Multiplex Oligo Set 1 index primers for Illumina chemistry were used. Final libraries were quantified using a Bioanalyzer DNA chip (Agilent) and subsequent qPCR. Each library was sequenced using paired-end, 100 cycles run on a HiSEQ 2000 using V3 sequencing chemistry. Each library was sequenced on 50-100% of a lane to result in a 6-9 median depth of coverage. Whole genome sequencing FASTQ files were aligned to the human genome (hg19) using Burrows-Wheeler Aligner (BWA) [[40](#_ENREF_40)] and copy number variations were identified using the Patchwork [[41](#_ENREF_41)] and GISTIC algorithms [[42](#_ENREF_42)] and annotated using build 37 of the genome.

**Phylogenetic Analysis**

Variants called by GATK were filtered to remove calls outside of the target capture regions and further filtered to remove calls where the depth of coverage in the MCF10A sample was ≤ 20 reads. The remaining variants that were not detected in the MCF10A (i.e. appeared *de novo* in one or more of the cell lines other than the parental MCF10A) were used in the tree construction. Where a variant was entirely absent from a cell line it was specified with a zero. Where a variant was detected in a cell line, it was specified with a 1. In addition to variants, copy number alterations of chromosomal arms were examined. Copy number loss and gain events were treated separately, i.e. Where a given arm of a chromosome was found to undergo loss in some cell lines but gain in other, these events were classified as two independent features where a given cell line could either have a gain even or not or could have a loss event or not. The set of informative features representing variants and copy number alterations that varied across cell lines in the series were used to construct a phylogenetic tree. The Manhattan distances between each pair of cell lines were calculated to produce a matrix summarising the number of differences across the informative features between every pair of cell lines. The Neighbour-joining method was used to construct a tree from the distances and this was represented as an unrooted tree (Figure 1). The 'ape' R package was used for tree construction and visualisation.

**Paired-end massively parallel RNA sequencing**

RNA sequencing was performed using 100ng of ribosomal-depleted RNA from cell lines grown on plastic (2D and as spheroids). Directional RNA libraries were prepared using the NEBNext Ultra Directional RNA Library Preparation kit according to the manufacturer’s instructions. Size selection for 200bp fragments was used, together with 8 cycles of PCR amplification and samples indexed as described above. Four indexed samples were pooled at equimolar concentrations and sequenced on a single lane of a HiSeq 2500 using SBS v3 chemistry (Illumina) (2 x 76 cycles) as described, to result in a minimum of 40 million paired-end reads. RNA sequencing FASTQ files were aligned to the human genome (hg19 build 37) using TopHat version 2.0.8b [[45](#_ENREF_45)]. Reads mapping to two or more locations were removed from the analysis. This resulted in an average of 44.5 million reads per sample. Data were visualised using the Integrative Genomics Viewer [[80](#_ENREF_80)]. Raw counts of reads mapped to genes were calculated using HT-Seq (http://www-huber.embl.de). These were used as input for differential gene expression analysis using DESeq2, with an adjusted P-value cutoff of <= 0.01 [[46](#_ENREF_46)]. The gene expression of the pre-invasive cells, (MCF10A, MCF10A1 and MCF10neoT) were compared with the invasive cells (MCF10Ca1a, MCF10Ca1d and MCF10Ca1h). MCF10DCIS.com were omitted from this analysis given that they form pre-invasive lesions *in vivo* that spontaneously become invasive [[47](#_ENREF_47)]. Fusion genes were identified using Chimerascan [[48](#_ENREF_48)] and deFuse [[49](#_ENREF_49)] algorithms. Pathway enrichment was performed using ConsensusPathDB [[50](#_ENREF_50)].

**RT-qPCR and Sanger sequencing validation**

Reverse transcription was performed with Superscript III (Invitrogen) using 50ng of RNA per reaction. Nominated in-frame fusion genes were screened by RT-PCR with primers spanning the nominated breakpoints using KAPA Taq DNA Polymerase (KAPA Biosystems, MA, USA) and sequenced as described [[51](#_ENREF_51)]. Sequences were visualised using 4Peaks (http://nucleobytes.com/4peaks/) In-frame fusion genes were quantitated in the cell line series using quantitative RT-PCR, with Power SYBR Green reagents (Life Technologies), in 96 well plate format on the ABI 9700HT. The relative abundance of the fusion transcript to β-actin was calculated using the delta-delta CT method. Primer sequences are listed in Supplementary Table S1.

**Western blotting**

Standard western blotting was conducted as previously described [63]. Antibodies against phospho-Akt (#4060) and total Akt (#2920) were purchased from Cell Signaling Technology. Quantification of conjugated secondary antibodies and analysis were performed using the Image Studio Software from LI-COR (LI-COR Biosciences).

**siRNA screen**

Genes were chosen to be screened in 96-well spheroid assays, based on either the i) the presence of either recurrent amplifications and homozygous deletions, or ii) non-synonymous coding mutations in the progression series. Alterations were chosen that were also present in primary tumours assessed from METABRIC [[52](#_ENREF_52)] and microarray comparative genomic hybridisation studies [[53-55](#_ENREF_53)] for copy number alterations and from TCGA and other published studies at a frequency >0.5% for somatic mutations [[56-60](#_ENREF_56)]. For amplifications and homozygous deletions, those that are either known drivers or predicted drivers (for amplifications) as assessed from a significant correlation of amplification with gene expression [[55](#_ENREF_55)] were triaged. For mutations, those that are known to be drivers or predicted to be drivers from the prediction algorithm FATHMM [[61](#_ENREF_61)] were triaged for functional assessment.

**Three-dimensional spheroid cultures**

For 3D siRNA transfections, 5000 cancer cells per well of a 96-well low attachment plate (Corning) were reverse-transfected with 37.5 nM of siGENOME smartpool siRNA (GEHealthcare) or siControls (positive ubiquitin B and negative (pool 1)) using Lullaby reagent (Oz biosciences) in 180 µl of cold culture medium as described [[62](#_ENREF_62)]. Low attachment plates were then centrifuged for 10 min at 800 x g (Sorvall Centrifuge). After 24 h, an additional 100 µl culture medium was added to each well of the 96-well plate. Spheroid area was calculated using the Celigo S (Nexcelom, MA, USA) and viability was measured using the CellTiter-Glo® assay (Promega, UK). Assays were terminated at the times indicated. Relative growth was calculated relative to siControl. Hits were scored as >1.2 for increased spheroid growth and <0.8 for reduced cell growth. Homozygous deletions were triaged out if there was no correlation of loss of expression with deletions. Hits were deconvoluted with individual oligos and validated if >2 individual oligos showed the same phenotype as the pool. siRNA-mediated knockdown was validated using qRT-PCR using Taqman probes (Life Technologies), or Sybr Green using Quanitect assays (Qiagen) or custom designed primers (Supplementary Table S1). Experiments were performed in triplicate.

**Transfections of mammalian cells on plastic**

For 2D siRNA transfections, 2500 cancer cells per well of a 96-well plate (Greiner) were reverse-transfected with 37.5 nM of Dharmacon siGENOME siRNA using Lullaby reagent (Oz biosciences). After 24 h, an additional 100 µl culture medium was added to each well. Cell viability was assessed 6-8 d post transfection as described, using the CellTiter-Glo® assay (Promega, UK). The cell survival fraction for each siRNA was calculated relative to non-targeting controls as described [[54](#_ENREF_54)]. Experiments were performed in triplicate.

**Immunohistochemistry of spheroid cultures**

Spheroids were grown for 7 d and fixed in 3.8% formaldehyde for 30 min, washed with PBS three times and stored at 4 °C. Spheroids were then pooled, dehydrated, embedded in paraffin and sectioned. The spheroid sections were then de-paraffinised with xylene, rehydrated, microwaved and then incubated overnight with primary antibodies. Staining was visualised using 3,3'-diaminobenzidine (DAB) secondary antibody. Ki67, (Dako, MIB-1 F726801-8, pressure cooker, pH6 citrate 2 min at 127 ^0^C, diluted 1/300); TP53, Dako, DO7, pressure cooker, pH6 citrate 2 min at 127 ^0^C diluted 1/200 and pAKT (Cell Signaling D9E pH 9.0, Dako pretreatment module 97 ^0^C for 20 min diluted 1/30).
