# Supplementary figures and images for: Three‐dimensional modelling identifies novel genetic dependencies associated with breast cancer progression in the isogenic MCF10 model
Source: J Pathol. 2016 Oct 19;240(3):315–28. doi: 10.1002/path.4778 (PMC5082563; doi:10.1002/path.4778)

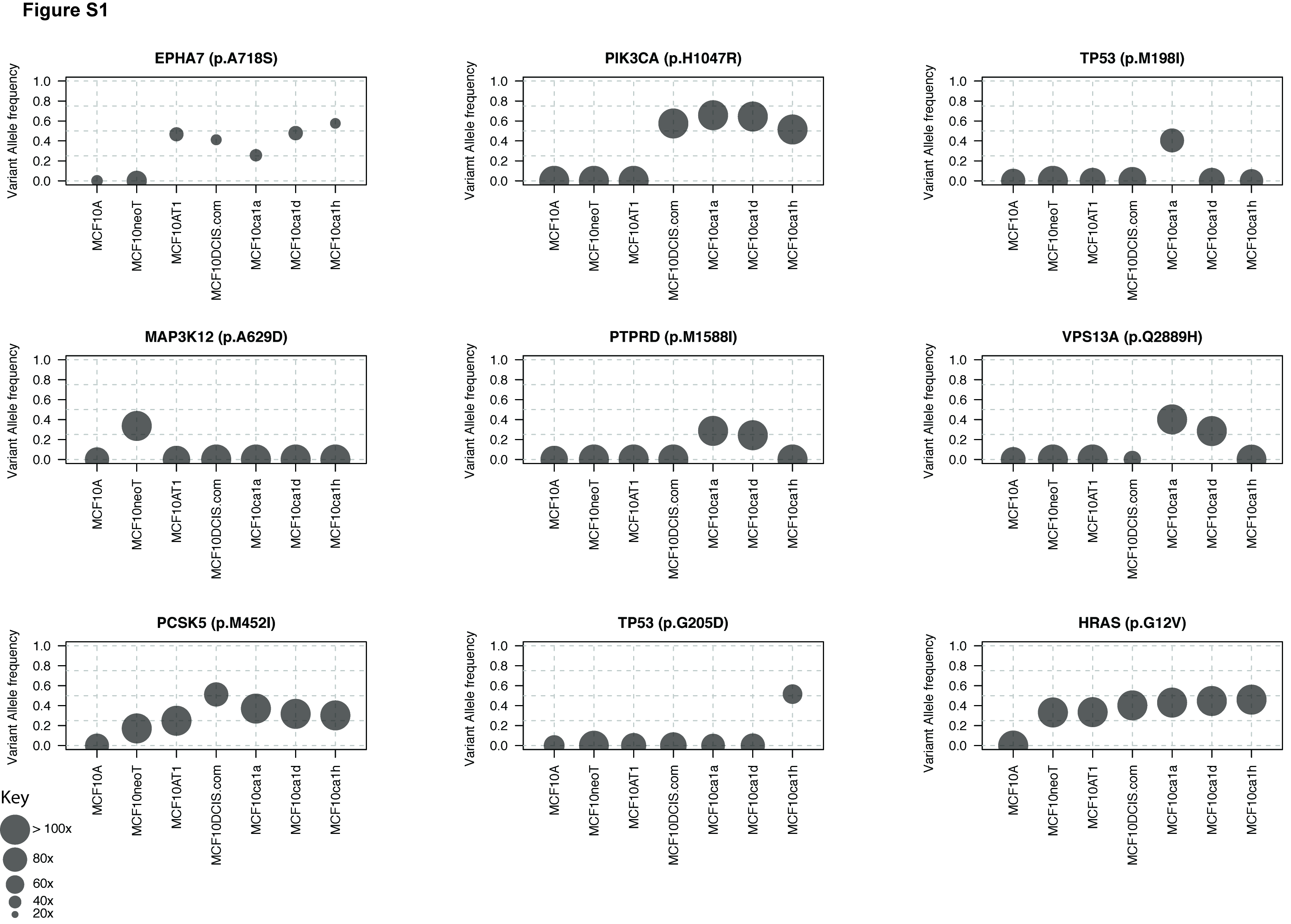

Supplement: Supplementary file 3 — Figure S1: Variant allele frequency plots of acquired mutations Variant allele frequency (VAF) plots of predicted driver mutations acquired in malignant cells, depicting the cell line (x‐axis) and VAF (y‐axis). The size of the dots corresponds to the depth of coverage. [file PATH-240-315-s004.tif]

Figure S2

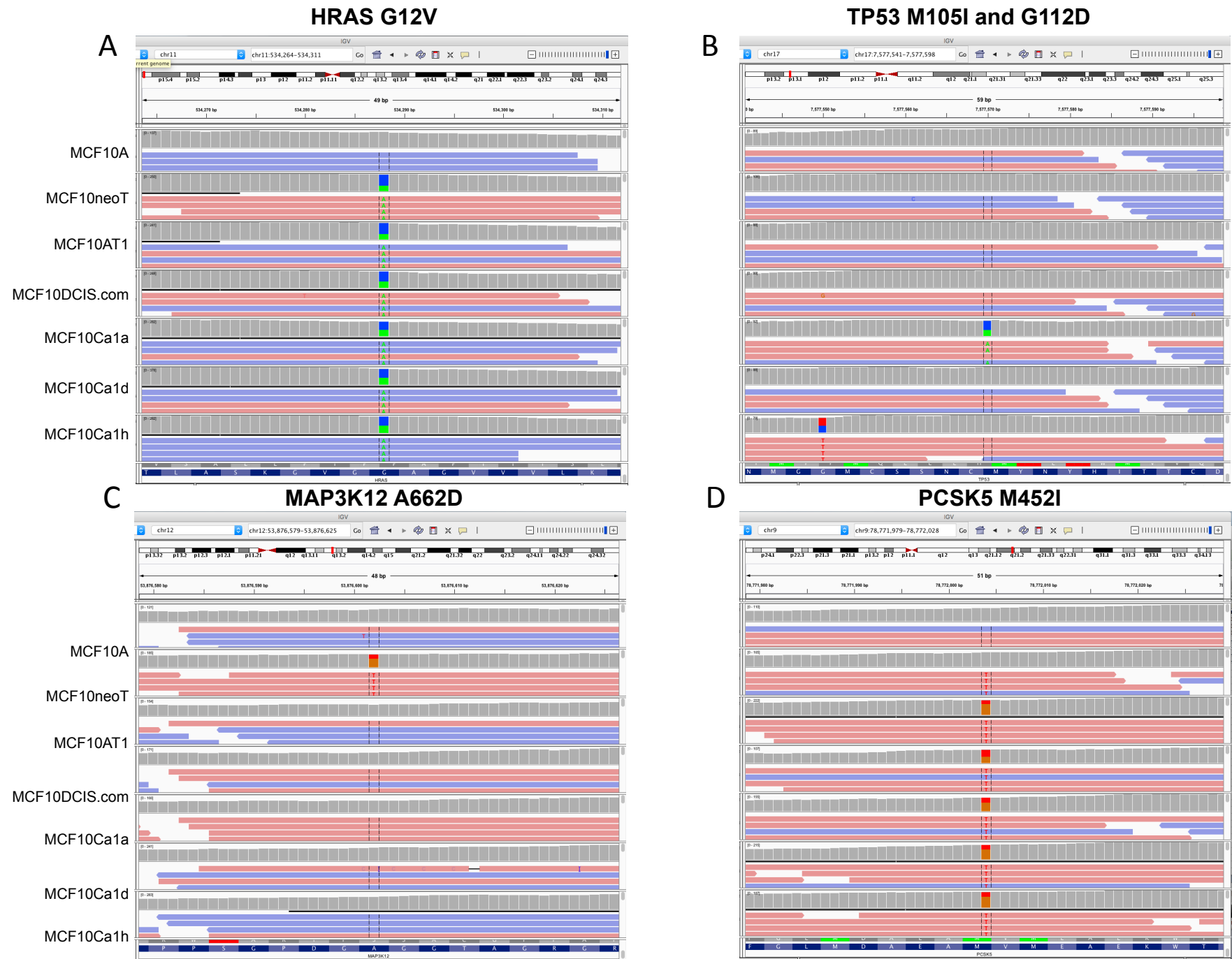

## PIK3CA H1047R

E

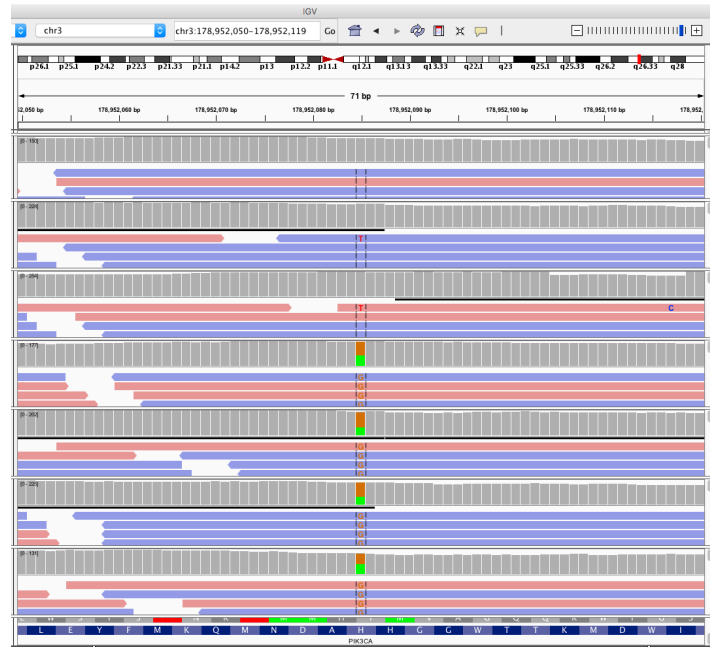

## PTPRD M118I

F

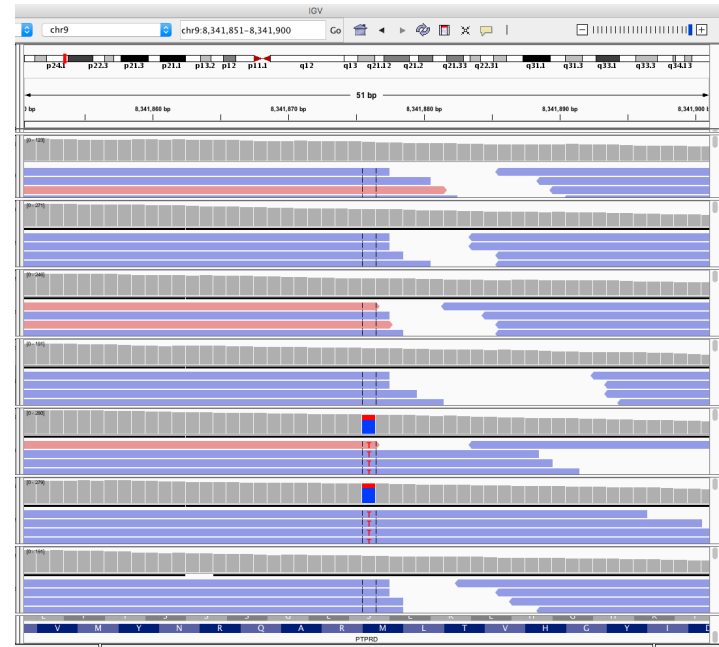

## VPS13A Q2850H

G

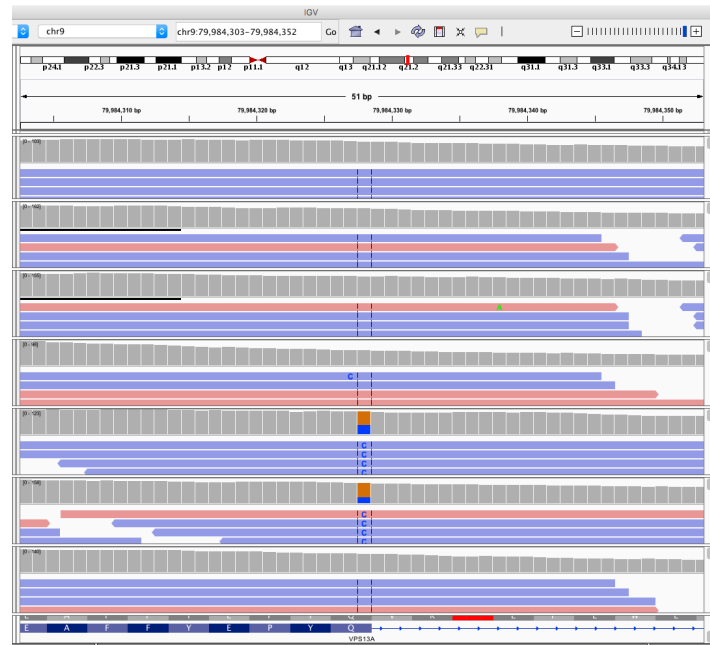

## EPHA7 A713S

H

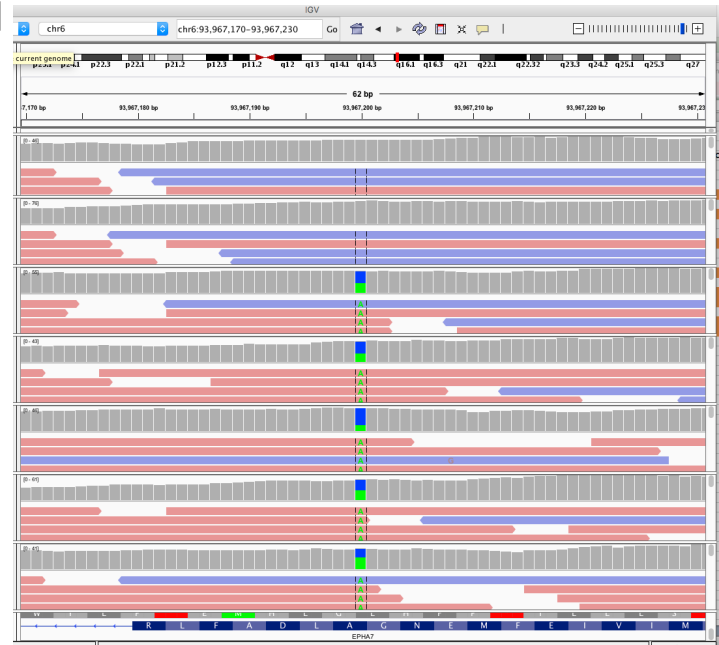

Supplement: Supplementary file 4 — Figure S2: IGV screenshots of acquired mutations in the MCF10 progression series Screenshots from Integrative Genomics Viewer (IGV) of driver genes acquired from in malignant cells, showing the presence or absence of mutant reads in the cell lines. Reads are sorted according to mutant reads. [file PATH-240-315-s013.pdf]

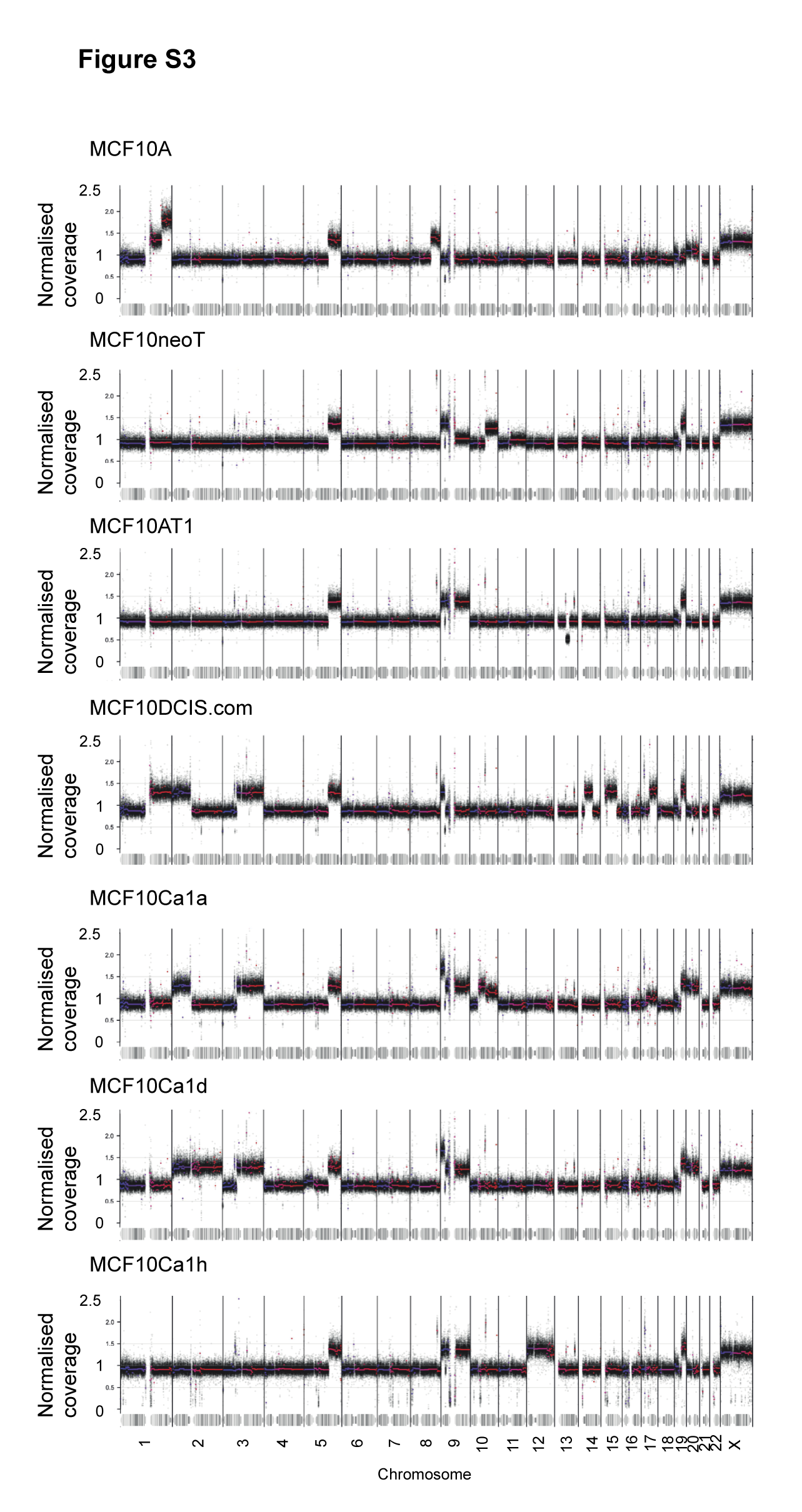

Supplement: Supplementary file 5 — Figure S3: Copy number alterations in the MCF10 progression series Genome plots showing normalised coverage of reads (y‐axis) from whole genome sequencing are plotted according to the genomic position in the genome (x‐axis). [file PATH-240-315-s011.tif]

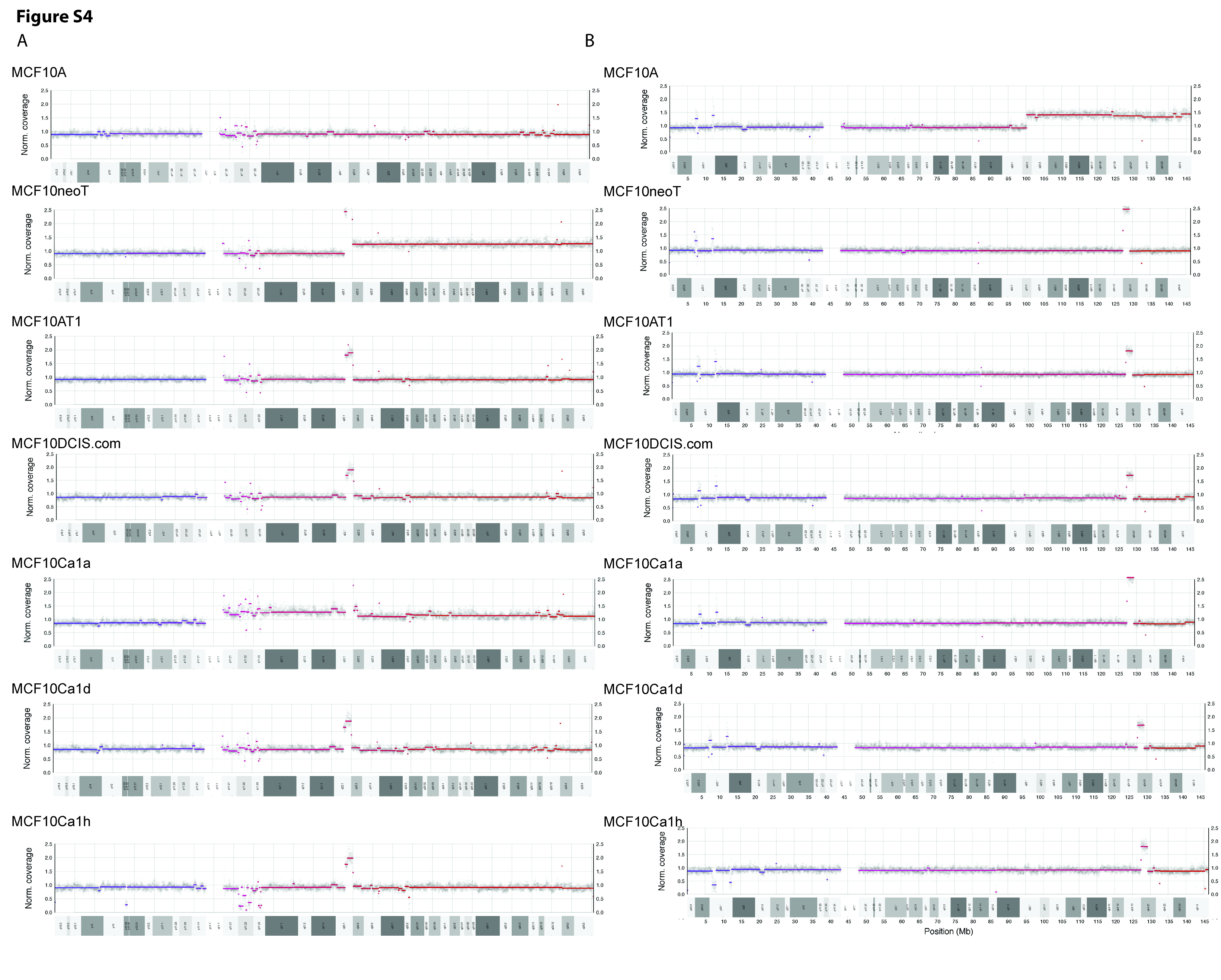

Supplement: Supplementary file 6 — Figure S4: Focal amplifications in the MCF10 progression series (A) Chromosome 10 and (B) Chromosome 17 plots showing focal amplifications acquired during progression. Normalised coverage (y‐axis) is plotted according to genomic position (x‐axis) [file PATH-240-315-s005.tif]

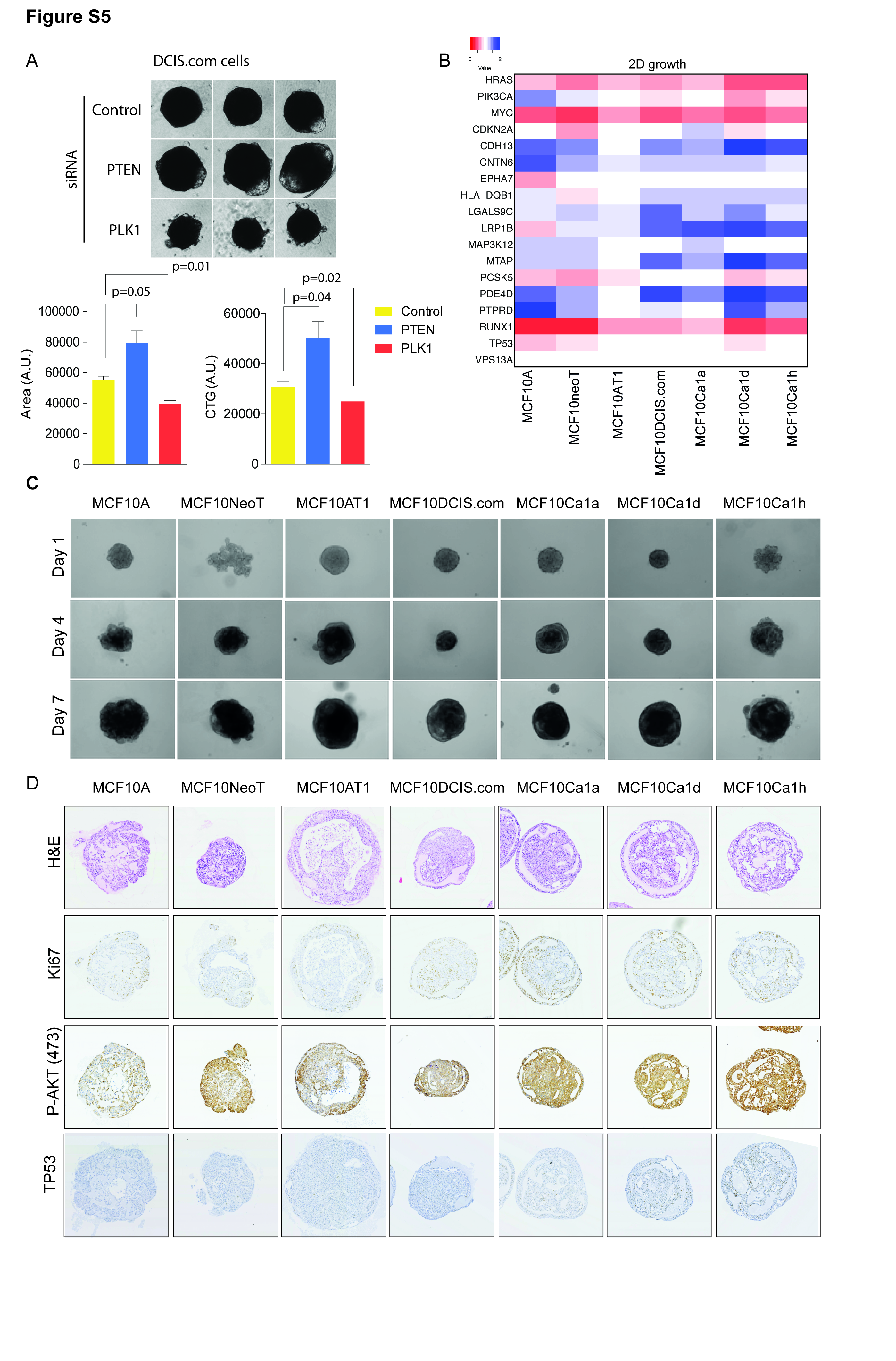

Supplement: Supplementary file 7 — Figure S5: RNAi modulation of the isogenic MCF10 model in cancer cell line spheroids (A) Micrograph images of MCF10DCIS.com cells subsequent to transfection of siRNA. Images were taken at day 7. (B) Heatmap of spheroid growth after siRNA mediated silencing and gene expression on plastic (2D). Relative spheroid growth is measured by the survival fraction of treated cells relative to siControl. (C) Micrograph images of spheroids at day 1, 4 and 7 of the MCF10 progression series, demonstrating good growth kinetics. (D) Images of spheroids stained with H&E and antibodies against Ki67, phosphor‐AKT, and TP53. [file PATH-240-315-s009.tif]

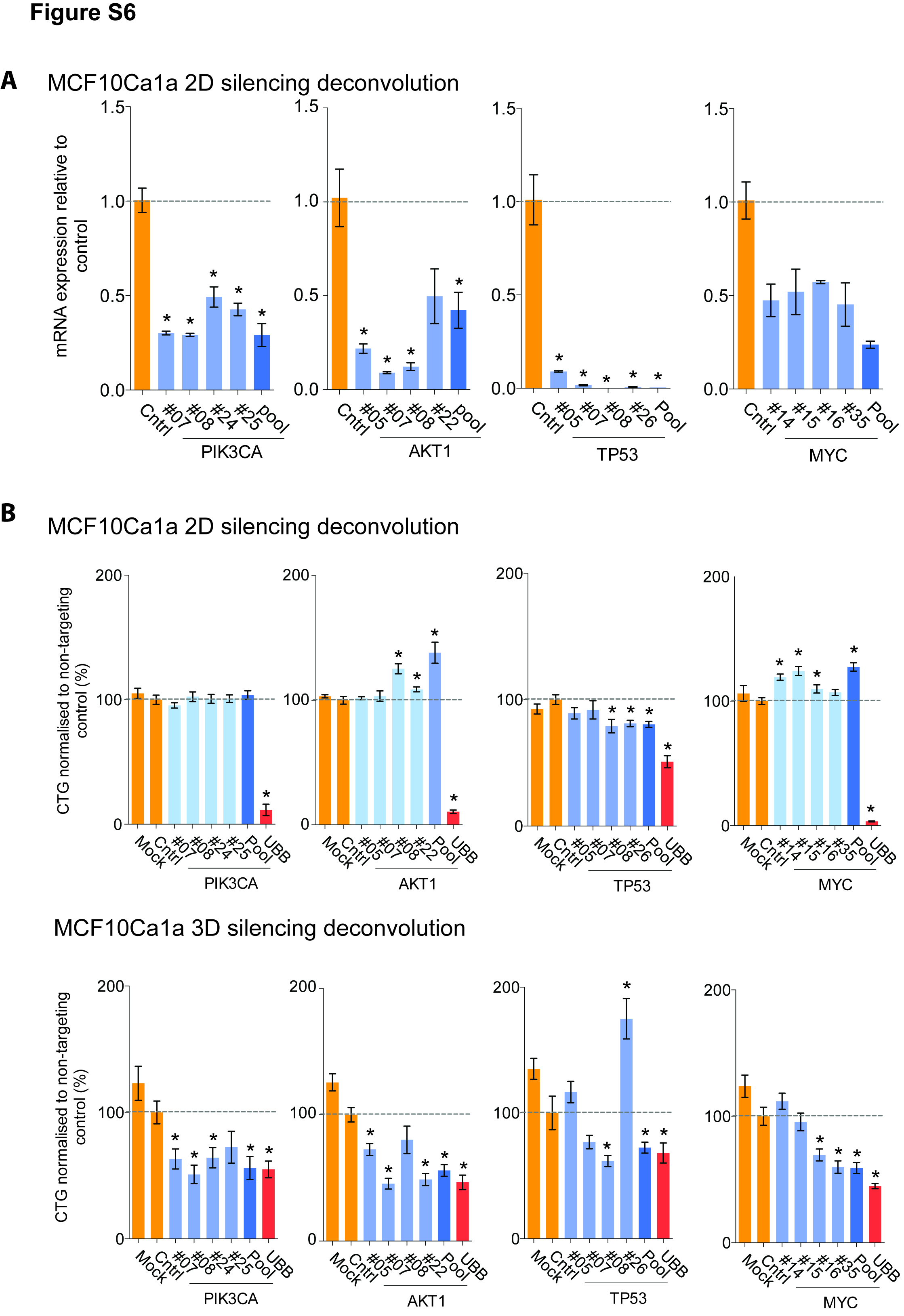

Supplement: Supplementary file 8 — Figure S6: Oligo deconvolution in MCF10Ca1a cells in spheroids and on plastic. (A) Barplots showing relative mRNA levels after gene silencing. Statistically significant alterations in mRNA expression were calculated using Student's t test (*p ≤ 0.05) (B) Barplots of relative cell growth in 2D and as 3D spheroids with individual oligos. Relative spheroid growth is measured by the survival fraction of treated cells relative to siControl. Statistical comparisons were performed using Student's t‐test (*p ≤ 0.05) [file PATH-240-315-s007.tif]
